# The impact of the final HERA combined data on PDFs obtained from a global fit

**DOI:** 10.1140/epjc/s10052-016-4020-1

**Published:** 2016-04-06

**Authors:** L. A. Harland-Lang, A. D. Martin, P. Motylinski, R. S. Thorne

**Affiliations:** 1 Department of Physics and Astronomy, University College London, London, WC1E 6BT UK; 2 Institute for Particle Physics Phenomenology, Durham University, Durham, DH1 3LE UK

## Abstract

We investigate the effect of including the HERA run I + II combined cross section data on the MMHT2014 PDFs. We present the fit quality within the context of the global fit and when only the HERA data are included. We examine the changes in both the central values and the uncertainties in the PDFs. We find that the prediction for the data is good, and only relatively small improvements in $\chi ^2$ and changes in the PDFs are obtained with a refit at both NLO and NNLO. PDF uncertainties are slightly reduced. There is a small dependence of the fit quality on the value of $Q^2_{\min }$. This can be improved by phenomenologically motived corrections to $F_L(x,Q^2)$ which parametrically are largely in the form of higher-twist type contributions.

## Introduction

The MSTW2008 PDFs [1] have been widely used in the analyses of hadron collider data. They were recently updated with an analysis performed in the same general framework, resulting in the MMHT2014 PDFs [2], and accompany recent updates by other groups [3–6], with the CT, MMHT and NNPDF sets having been combined in an updated PDF4LHC recommendation [7]. The MMHT 2014 PDFs were an improvement to the MSTW 2008 PDFs partially due to a number of developments in the procedures employed in the analysis. For example, we now use modified and extended parameterisations for the PDFs based on Chebyshev polynomials, and we allow freedom in the deuteron nuclear corrections, both these features being introduced in [8]. This led to a change in the $u_V$–$d_V$ distribution and an improved description of the LHC data for the *W* boson charge asymmetry. Additionally, we now use the “optimal” GM-VFNS choice [9] which is smoother close to heavy flavour transition points, particularly at NLO. The correlated systematic uncertainties, which are important for jet data in particular, are now treated as multiplicative rather than additive. We have also changed the value of the charm branching ratio to muons used to $B_{\mu } = 0.092$ and allow an uncertainty of $\pm 10~\%$ [10]. This feeds into the central value and the uncertainty of the strange quark PDF.

There are also a wide variety of new data sets included in the MMHT fit. These include *W*, *Z* cross sections from ATLAS, CMS and LHCb, differential in rapidity; Drell Yan data at high and low mass; and also data on $\sigma _{t\bar{t}}$ from the Tevatron and from ATLAS and CMS. At NLO we also include ATLAS and CMS inclusive jet data from the 7 TeV run, though we do not yet include these data at NNLO. Previous analyses have used threshold corrections for the Tevatron jet data, and we continue to include these data in the NNLO analysis. However, for jet data from the LHC we are often far from threshold, and the approximation to the full NNLO calculation is not likely to be reliable. The full NNLO calculation [11, 12] is nearing completion. There are also various changes in non-LHC data sets, for example we include some updated Tevatron *W* boson asymmetry data sets. The single most important change in data included is the replacement of the HERA run I neutral and charged current data provided separately by H1 and ZEUS with the combined HERA data set [13] (and we also include HERA combined data on $F_2^c(x,Q^2)$ [14]). These are the data which provide the best single constraint on PDFs, particularly on the gluon at all $x < 0.1$.

However, in [2] we decided not to include any separate run II H1 and ZEUS data sets since it was clear the full run I $+$ II combined data would soon appear. This has now recently happened, and the data, and the accompanying PDF analysis, are published in [15]. It was not stated in [2] precisely when an update of MMHT2014 PDFs would be required. Significant new LHC data would be one potential reason, and the full NNLO calculation of the jet cross sections, effectively allowing a larger data set at NNLO, might be another. The potential impact of the final HERA inclusive cross section data was another factor in this decision, it being possible that these alone might produce a very significant change in either the central value of the PDFs or their uncertainties, or both. Hence, it is now obviously a high priority to investigate their impact.^1^ However, as well as just investigating the impact of the new data on the PDFs assuming a standard fixed-order perturbative treatment, it is also interesting to investigate the quality of the fit, and to see if it is possible to improve the quality in some regions of *x* and $Q^2$. In particular, there is a suggestion in [15] that the data at low $Q^2$ are not fit as well as they could be, so we first confirm that we also see this feature, and we also investigate, in a very simple manner, what type of corrections can solve this problem.

## Fit to combined HERA data set

If we use our standard cut of $Q^2_{\min }=2~\mathrm GeV^2$ to eliminate data with $Q^2$ below this value, there are 1185 HERA data points with 162 correlated systematics and 7 procedural uncertainties. These are naturally separated into 7 subsets, depending on whether the data are obtained from $e^+$ or $e^-$ scattering from the proton, whether it is from neutral or charged current scattering, and on the proton beam energy $E_p$. This is to be compared to 621 data points, separated into 5 subsets, with generally larger uncertainties, from the HERA I combined data used previously (though these data do have fewer correlated systematics). We first investigate the fit quality from the predictions using MMHT2014 PDFs and without performing any refit. We use the same $\chi ^2 \mathrm{definition}$ as in [2], i.e.

1 $$\begin{aligned} \chi ^2=\sum _{i=1}^{N_\mathrm{pts}}\left( \frac{D_i+\sum _{k=1}^{N_\mathrm{corr}} r_k\sigma _{k,i}^\mathrm{corr}-T_i}{\sigma _i^\mathrm{uncorr}}\right) ^2+\sum _{k=1}^{N_\mathrm{corr}}r_k^2, \end{aligned}$$

where $D_i+\sum _{k=1}^{N_\mathrm{corr}}r_k\sigma _{k,i}^\mathrm{corr}$ are the data values allowed one to shift by some multiple $r_k$ of the systematic error $\sigma _{k,i}^\mathrm{corr}$ in order to give the best fit, and where $T_i$ are the parametrised predictions. The results obtained are already rather good:

$$\begin{aligned}&\chi ^2_\mathrm{NLO} = 1611/1185 = 1.36 \,\mathrm{per point}.\\&\chi ^2_\mathrm{NNLO} = 1503/1185 = 1.27 \,\mathrm{per point}. \end{aligned}$$

This is to be compared to the result in [15] with HERAPDF2.0 PDFs, which are fit to (only) these data. They obtain $\sim$1.20 per point using $Q^2_{\min }=2~\mathrm GeV^2$, at both NLO and NNLO. Hence, we do not expect dramatic improvement to the fit quality from our predictions by refitting, particularly at NNLO. Next we perform a refit in the context of our standard global fit, i.e. we simply replace the previous HERA run I data with the new run I $+$ II combined data. There are no procedural changes to the fit at all. The fit quality improves toThis is a significant, but hardly dramatic improvement (and much less than the improvement after refitting when HERA run I combined data were first introduced into the MSTW2008 fitting framework [18]), i.e. the MMHT2014 PDFs are already giving quite close to the best fit within the global fit framework.

**Table 1:** The $\chi ^2$ for each subset of HERA I + II data for our four different fits with $Q^2_{\min }=3.5~\mathrm GeV^2$. Note that this data cut eliminates 40 HERA data points as compared to fit with $Q^2_{\min }=2~\mathrm GeV^2$. In this table the $\chi ^2$ per data set does not include the penalties for shifts in systematic parameters, which is separated out at the top of the table. This is the only place in the article where this separation has been made

	No. points	NLO $\chi ^2_\mathrm{HERA}$	NLO $\chi ^2_\mathrm{global}$	NNLO $\chi ^2_\mathrm{HERA}$	NNLO $\chi ^2_\mathrm{global}$
Correlated penalty		79.9	113.6	73.0	92.1
CC $e^+p$	39	43.4	47.6	42.2	48.4
CC $e^-p$	42	52.6	70.3	47.0	59.3
NC $e^-p$ $E_p=920~\mathrm GeV$	159	213.6	233.1	213.5	226.7
NC $e^+p$ $E_p=920~\mathrm GeV$	377	435.2	470.0	422.8	450.1
NC $e^+p$ $E_p=820~\mathrm GeV$	70	67.6	69.8	71.2	69.5
NC $e^-p$ $E_p=575~\mathrm GeV$	254	228.7	233.6	229.1	231.8
NC $e^-p$ $E_p=460~\mathrm GeV$	204	221.6	228.1	220.2	225.6
Total	1145	1342.6	1466.1	1319.0	1403.5

In order to compare more directly with the HERAPDF2.0 study we also fit to only HERA run I $+$ II data. This requires us to fix four of our normally free PDF parameters in order to avoid particularly unusual PDFs. In practice the danger is a very complicated, and potentially pathological, strange quark distribution, which can fluctuate dramatically as HERA data do not have any direct constraint on the *s* and ${\bar{s}}$ PDFs. We allow the $s + \bar{s}$ distribution to have a free normalisation and high-*x* power but all other shape freedom is removed. The *s*–$\bar{s}$ asymmetry is fixed to the MMHT2014 default value. With these restrictions, the result of our fit is

$$\begin{aligned}&\chi ^2_\mathrm{NLO} = 1416/1185 = 1.19\, \mathrm{per\, point }\\&\chi ^2_\mathrm{NNLO} = 1381/1185 = 1.17\, \mathrm{per\, point } \end{aligned}$$

Hence, in this case, as well as the global fit, the NNLO fit quality is still definitely better than that at NLO, but not as distinctly.

We also perform the fit with $Q^2_\mathrm{min}=3.5~\mathrm GeV^2$ in order to compare in detail with the results in [15], where this is their default cut. In Table 1 we show the breakdown of $\chi ^2$ values for the different HERA neutral and charged current data sets. We include the numbers for the global fit including the HERA combined data, as well as the results for the fit to the HERA data only, at both NLO and NNLO. There appears to be some tension between the $e^- p$ charged current data and other data in the global fit, with the NLO fit to the HERA only data giving a $\chi ^2$ for these data which is $\sim$20 units higher than the global fits. The tension is somewhat lower at NNLO, where the increase is $\sim$10 units less. The $\chi ^2$ for the neutral current data at 920 GeV also shows some, albeit relatively lower, sensitivity to whether a global fit is performed.

**Fig. 1:**
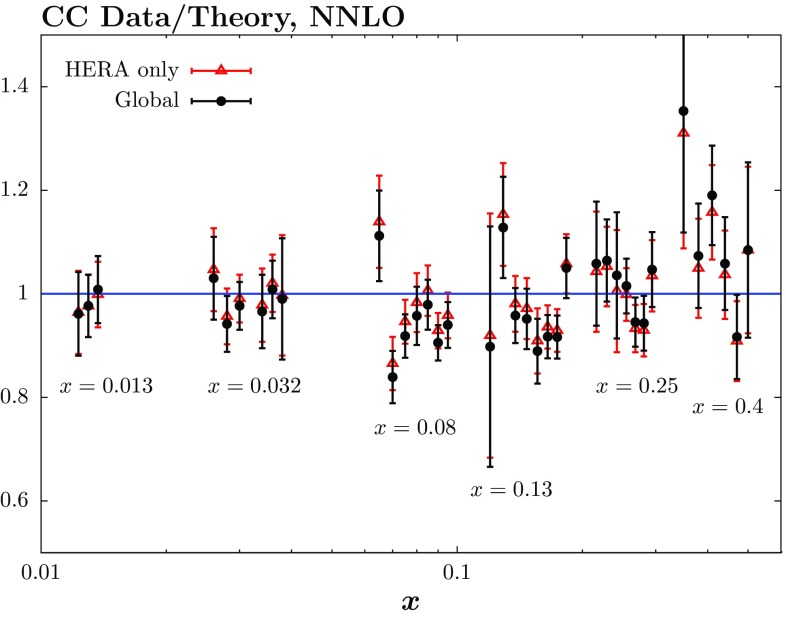
HERA $e^-$ charged current data divided by theory for the local fit to HERA II combined data, and for the global fit including this data set. The shifts of data relative to theory due to correlated uncertainties are included. The data are shown at different values of *x*, as indicated on the *plot*

In Fig. 1 we show the data/theory at NNLO for the $e^-$ charged current data in different *x* bins. It can be seen that while the local fit gives a good description of the data, the comparison for the global fit has a different shape. It tends to largely overshoot the data at intermediate *x*, i.e. in bins $x=0.032, 0.08, 0.13$, but generally undershoots it at higher *x*. These charged current data are mainly sensitive to the up (at high *x* valence) quark. Hence, in the global fit data other than HERA data, in practice largely fixed proton target DIS data, clearly prefer a different shape for the up quark. In particular, the HERA charged current data prefers a somewhat smaller/larger *u* quark at intermediate/larger *x* compared to the other global data. We will return to this in the next section.

## Effect on the PDFs

**Fig. 2:**
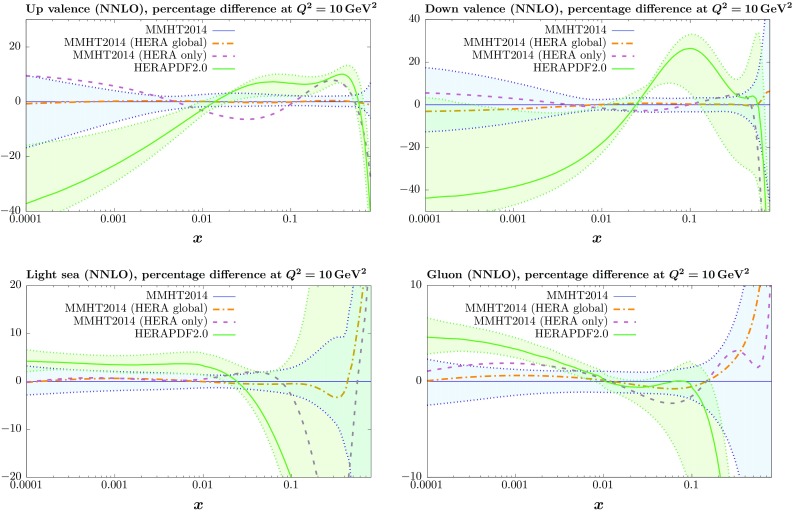
Comparison between the up and down valence, gluon and light quark sea distributions at $Q^2=10^4~\mathrm GeV^2$ for the standard MMHT2014 fit, with the corresponding PDF uncertainties, with the central values of the fit including the HERA combined data, as well as the fit to only this data set, shown as *dot-dashed* and *dashed curves*, respectively. Also shown are the HERAPDF2.0 distributions, including PDF uncertainties

Since the fit quality does not improve very significantly from the prediction using the MMHT 2014 PDFs we do not expect much change in the central value of the PDFs in the new global fit which includes the HERA I + II combined data. More change might be expected in the PDFs fit to only HERA data as then the main constraints on some types of PDF are lost. In Fig. 2 we show the central values of the NNLO PDFs from the fits including the new HERA combined data, comparing them to MMHT2014 PDFs (with uncertainties) and the HERAPDF2.0 PDFs (also with uncertainties). The modified global PDFs are always very well within the MMHT2014 uncertainty bands.

The PDFs from the fit to only HERA run I $+$ II data are in some ways similar to those of HERAPDF2.0, e.g. the up valence quark for $x>0.2$, which shows some significant deviations from the global fits PDF set. This appears to be driven by the $e^-$ charged current data, but there is clearly tension with the rest of the data in the global fit, as our full fit including the new HERA data does not have this feature. Similarly, the sea quarks in our fit to only HERA data prefer to be soft at high *x*, like for HERAPDF2.0, but in this case there is no real constraint on high-*x* sea quarks from HERA DIS data, and the HERAPDF2.0 uncertainty band is not in conflict with the global fits. However, the common features between our fit to only HERA run I $+$ II data and HERAPDF2.0 are not universal – the gluon and the down valence distributions in our fit to only HERA data are much more similar to MMHT2014 than HERAPDF2.0. This is likely to be a feature of the differing parameterisations used in the two studies. The very high-*x* gluon in the global fits definitely prefers a harder gluon than in HERAPDF2.0, due to constraints from jet data and fixed target DIS data, but even in our HERA data only fit, there is no actual preference for the softer high-*x* gluon. Also, we certainly see no suggestion of HERA data preferring a significantly different shape down valence distribution to that preferred by other sets in the global fit, and our central value in the HERA data only fit is surprisingly close to that in our global fits given the relative lack of constraint on this distribution from HERA DIS data.

**Fig. 3:**
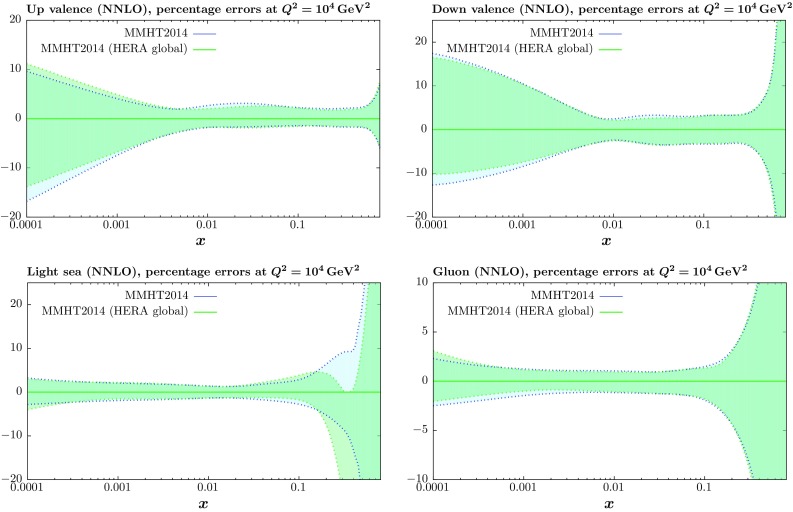
Comparison between the up and down valence, gluon and light quark sea distributions at $Q^2=10^4~\mathrm GeV^2$ for the MMHT2014 set and the corresponding uncertainties and the fit including the HERA combined data set with their corresponding uncertainties

We also investigate the effect of the new HERA data on the uncertainties of the PDFs. In order to determine PDF uncertainties we use the same “dynamic tolerance” prescription to determine eigenvectors as for MSTW2008 [1]. In Fig. 3 we compare the uncertainties for the NNLO PDFs including the HERA run I $+$ II data in a global fit to the uncertainties of the MMHT2014 PDFs. These are very similar to MMHT2014 in most features. The most obvious improvement from the inclusion of the new HERA data is to the gluon for $x < 0.01$. There is also a slight improvement in some places for the valence quarks, but the additional constraint supplied by much improved charged current data is overwhelmed by the constraint of valence quark PDFs from other data in the global fit. While the improvements generally appear to be quite moderate, in fact when benchmark cross section predictions are considered, the effect of the HERA combined data in reducing the corresponding PDF uncertainties becomes somewhat clearer; we consider this in the following section.

**Table 2:** The values of various cross sections (in nb) obtained with the NNLO MMHT 2014 sets, with and without the final HERA combination data set included. PDF uncertainties only are shown

	MMHT14	MMHT14 (HERA global)
$W\,\, \mathrm{Tevatron}\,\,(1.96~\mathrm TeV)$	$2.782^{+0.056}_{-0.056}$ $\left( {}^{+2.0~\%}_{-2.0~\%}\right)$	$2.789^{+0.050}_{-0.050}$ $\left( {}^{+1.8~\%}_{-1.8~\%}\right)$
$Z \,\,\mathrm{Tevatron}\,\,(1.96~\mathrm TeV)$	$0.2559^{+0.0052}_{-0.0046}$ $\left( {}^{+2.0~\%}_{-1.8~\%}\right)$	$0.2563^{+0.0047}_{-0.0047}$ $\left( {}^{+1.8~\%}_{-1.8~\%}\right)$
$W^+ \,\,\mathrm{LHC}\,\, (7~\mathrm TeV)$	$6.197^{+0.103}_{-0.092}$ $\left( {}^{+1.7~\%}_{-1.5~\%}\right)$	$6.221^{+0.100}_{-0.096}$ $\left( {}^{+1.6~\%}_{-1.5~\%}\right)$
$W^- \,\,\mathrm{LHC}\,\, (7~\mathrm TeV)$	$4.306^{+0.067}_{-0.076}$ $\left( {}^{+1.6~\%}_{-1.8~\%}\right)$	$4.320^{+0.064}_{-0.070}$ $\left( {}^{+1.5~\%}_{-1.6~\%}\right)$
$Z \,\,\mathrm{LHC}\,\, (7~\mathrm TeV)$	$0.964^{+0.014}_{-0.013}$ $\left( {}^{+1.5~\%}_{-1.3~\%}\right)$	$0.966^{+0.015}_{-0.013}$ $\left( {}^{+1.6~\%}_{-1.3~\%}\right)$
$W^+ \,\,\mathrm{LHC}\,\, (14~\mathrm TeV)$	$12.48^{+0.22}_{-0.18}$ $\left( {}^{+1.8~\%}_{-1.4~\%}\right)$	$12.52^{+0.22}_{-0.18}$ $\left( {}^{+1.8~\%}_{-1.4~\%}\right)$
$W^- \,\,\mathrm{LHC}\,\, (14~\mathrm TeV)$	$9.32^{+0.15}_{-0.14}$ $\left( {}^{+1.6~\%}_{-1.5~\%}\right)$	$9.36^{+0.14}_{-0.13}$ $\left( {}^{+1.5~\%}_{-1.4~\%}\right)$
$Z \,\,\mathrm{LHC}\,\, (14~\mathrm TeV)$	$2.065^{+0.035}_{-0.030}$ $\left( {}^{+1.7~\%}_{-1.5~\%}\right)$	$2.073^{+0.036}_{-0.026}$ $\left( {}^{+1.7~\%}_{-1.3~\%}\right)$
$\mathrm{Higgs} \,\,\mathrm{Tevatron}$	$0.874^{+0.024}_{-0.030}$ $\left( {}^{+2.7~\%}_{-3.4~\%}\right)$	$0.866^{+0.019}_{-0.023}$ $\left( {}^{+2.2~\%}_{-2.7~\%}\right)$
$\mathrm{Higgs} \,\,\mathrm{LHC}\,\,(7~\mathrm TeV)$	$14.56^{+0.21}_{-0.29}$ $\left( {}^{+1.4~\%}_{-2.0~\%}\right)$	$14.52^{+0.19}_{-0.24}$ $\left( {}^{+1.3~\%}_{-1.7~\%}\right)$
$\mathrm{Higgs} \,\,\mathrm{LHC}\,\,(14~\mathrm TeV)$	$47.69^{+0.63}_{-0.88}$ $\left( {}^{+1.3~\%}_{-1.8~\%}\right)$	$47.75^{+0.59}_{-0.72}$ $\left( {}^{+1.2~\%}_{-1.5~\%}\right)$
$t\bar{t} \,\,\mathrm{Tevatron}$	$7.51^{+0.21}_{-0.20}$ $\left( {}^{+2.8~\%}_{-2.7~\%}\right)$	$7.57^{+0.18}_{-0.18}$ $\left( {}^{+2.4~\%}_{-2.4~\%}\right)$
$t\bar{t}\,\,\mathrm{LHC}\,\,(7~\mathrm TeV)$	$175.9^{+3.9}_{-5.5}$ $\left( {}^{+2.2~\%}_{-3.1~\%}\right)$	$174.8^{+3.3}_{-5.3}$ $\left( {}^{+1.9~\%}_{-3.0~\%}\right)$
$t\bar{t}\,\,\mathrm{LHC}\,\,(14~\mathrm TeV)$	$970^{+16}_{-20}$ $\left( {}^{+1.6~\%}_{-2.1~\%}\right)$	$964^{+13}_{-19}$ $\left( {}^{+1.3~\%}_{-2.0~\%}\right)$

## Effect on benchmark cross sections

In Table 2 we show NNLO predictions for benchmark *W*, *Z*, Higgs and $t\overline{t}$ cross sections at a range of collider energies, for the standard MMHT14 PDF set, and for the result of the same fit, but including the HERA combined data.

To calculate the cross section we use the same procedure as was used in [2]. That is, for *W*, *Z* and Higgs production we use the code provided by Stirling, based on the calculation in [19, 20] and [21], and for top pair production we use the procedure and code of [22]. Here our primary aim is not to present definitive predictions or to compare in detail to other PDF sets, as both these results are frequently provided in the literature with very specific choices of codes, scales and parameters which may differ from those used here. Rather, our main objective is to illustrate the effect that the combined HERA data has on the central values and uncertainties of the cross sections.

For *W*, *Z* production the central values of the predicted cross sections are only slightly affected by the inclusion of the HERA data, while there is some small, i.e. up to a few $\%$ level, reduction in the PDF uncertainties. For Higgs Boson production the predicted cross sections again change very little – well within PDF uncertainties. However, here the reduction in PDF uncertainty is larger, up to $\sim$10 % of the MMHT uncertainty. Finally, for $t\overline{t}$ production the picture is similar to the Higgs case, with the central value relatively unchanged, and the uncertainties reduced at the $\sim$10 % level. This highlights that the new HERA data provides some extra constraint within the global fit, but mainly due to the reduced uncertainty on the gluon distribution for the LHC predictions.

**Fig. 4:**
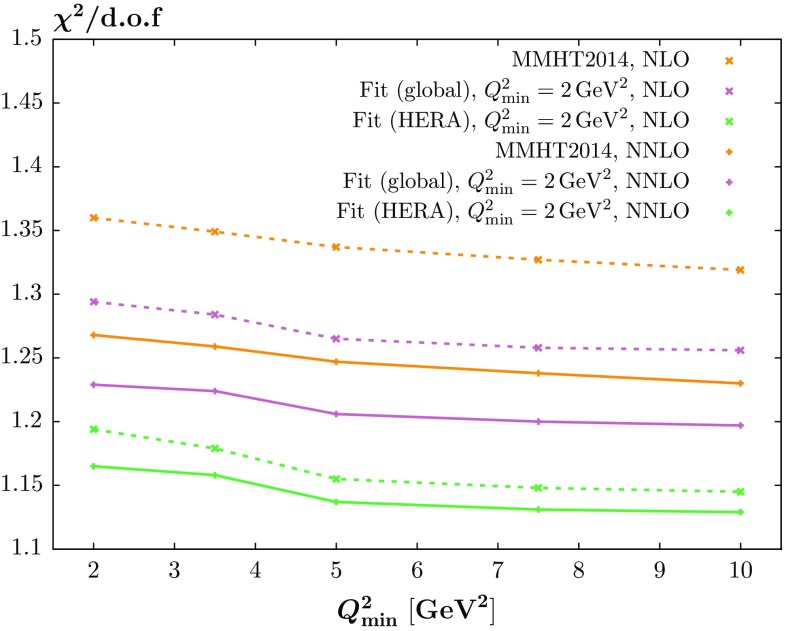
The $\chi ^2$ per degree of freedom for the MMHT2014 *predictions* (which occur in the plot in descending order) to the HERA combined data set, and for the global + HERA combined and HERA combined only fits, with $Q^2_\mathrm{min}=2\,\mathrm{GeV}^2$ fixed; the plot versus $Q^2_\mathrm{min}$ is then obtained by calculating the $\chi ^2$/d.o.f. for the HERA combined data with $Q^2>Q^2_{\min }$. The NLO (NNLO) curves are shown as *dashed* (continuous) *curves*

**Fig. 5:**
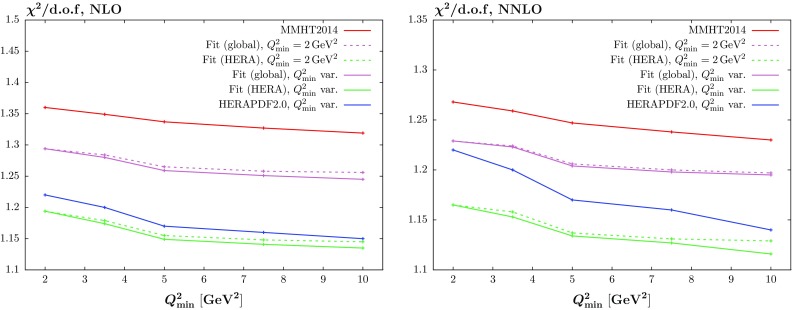
The $\chi ^2$ per degree of freedom for the MMHT2014 *predictions* to the HERA combined data set, and for the global + HERA combined and HERA combined only fits, with $Q^2_\mathrm{min}=2\,\mathrm{GeV}^2$; the plot versus $Q^2_\mathrm{min}$ is then obtained by calculating the $\chi ^2$ contribution from the HERA combined data with $Q^2>Q^2_\mathrm{min}$. These are shown (reproduced from Fig. 4) as *dashed curves*, while the two *solid curves* just below these show the effect of *fits* with $Q^2_{\min }$ varied (rather than fixed at $Q^2_{\min }=2~\mathrm GeV^2$). The result of the HERAPDF2.0 fit with varying $Q^2_\mathrm{min}$ is also shown. The *left/right hand figure* shows the NLO/NNLO fits

## Investigation of $Q^2_\mathrm{min}$ dependence

The HERAPDF2.0 analysis sees a marked improvement in $\chi ^2$ per point with a raising of the $Q^2_{\min }$ value for the data fit. Hence, we also investigate the variation of the fit quality for changes of $Q^2_{\min }$. However, to begin with we simply calculate the quality of the comparison to data as a function of $Q^2_{\min }$ at NLO and at NNLO without performing a refit, i.e. the PDFs used were those obtained with the default $Q^2_{\min }=2~\mathrm GeV^2$ cut. This is shown in Fig. 4 where we show a comparison of the $\chi ^2$ per point for the three variations of NLO and NNLO comparisons, i.e. the MMHT2014 prediction, the global refit including the new HERA data and the refit with only HERA run I $+$ II combined data. From the figure it is clear that NNLO is always superior, but this is less distinct in the refits, particularly for the fit to only HERA data. It is also clear there is a reasonable lowering of the $\chi ^2$ per point as $Q^2_{\min }$ increases, but no clear “jumps” in improvement.

We also look at the effect of changing the $Q^2$ cut in the fit itself (though we change the cut only for the HERA combined data, not for the other data in the global fit), at both NLO and NNLO. This is shown in Fig. 5, where we also show the trend for the HERAPDF2.0 analysis [15].^2^ For comparison we also include the curves from Fig. 4 for the $\chi ^2$ per point obtained for varying $Q^2_{\min }$ but with the fits performed for $Q^2_{\min }=2~\mathrm GeV^2$. We note that while there is an improvement in $\chi ^2$ per point with increasing $Q^2_{\min }$, as observed in [15], this is very largely achieved without any refitting. This is more marked in the global fit, where (at NNLO in particular) the refit with raised $Q^2_{\min }$ has only a minimal effect. It is very clear there is also less improvement with $Q^2_{\min }$ in our analysis than for HERAPDF2.0, particularly in the global fit and at NNLO. This may be due to our more extensive PDF parameterisation obtaining shapes that manage to fit the lowest $Q^2$ data better.

**Fig. 6:**
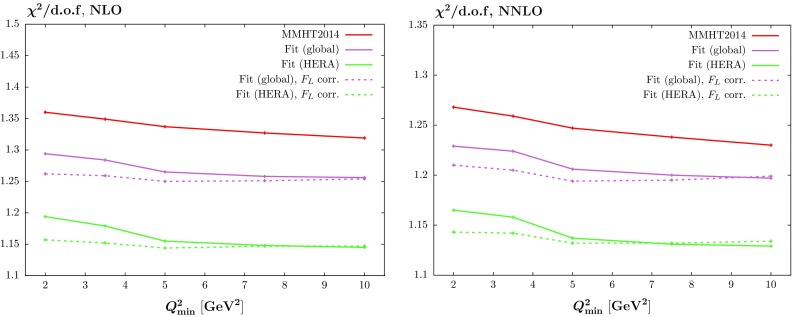
The behaviour of the $\chi ^2$ per degree of freedom when we include the higher-twist correction (3), shown by the *dashed curves*, as compared to the curves of Fig. 4 which were obtained without the correction. The *left/right hand figure* shows the NLO/NNLO fits

**Fig. 7:**
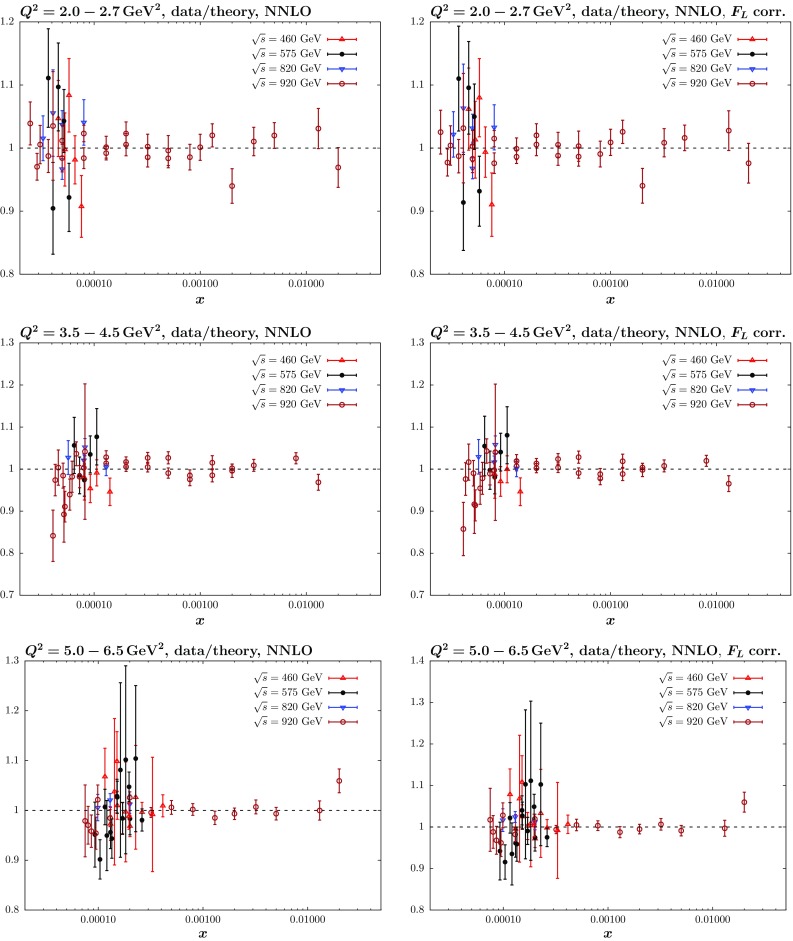
HERA NC data/theory for global MMHT fit including HERA combined data without (*left*) and with (*right*) the correction (3) applied, divided into individual data sets and for three ranges of $Q^2=2.0-2.7,\, 3.5-4.5,\, 5.0-6.5\,\mathrm{GeV}^2$. The shifts of data relative to theory due to correlated uncertainties are included

**Fig. 8:**
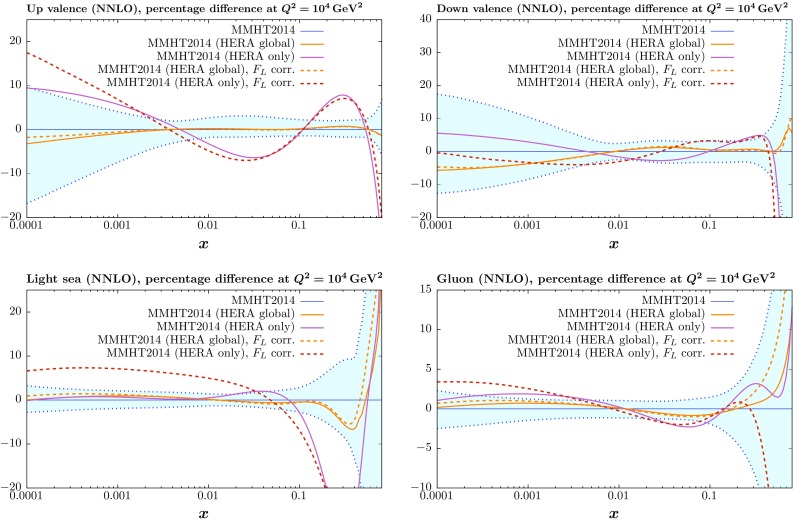
Comparison between the up and down valence, gluon and light quark sea distributions at $Q^2=10^4~\mathrm GeV^2$ for the standard MMHT2014 fit, with the MMHT2014 PDF errors, and for the central fits including the HERA combined data, as well as the fit to only this data set, with and without the correction (3) applied to $F_L$

## Effect of higher-twist type corrections

In order to investigate the possibility of improving the $\chi ^2$ per point for low $Q^2_{\min }$ we will consider some simple phenomenological corrections to the reduced cross section

2 $$\begin{aligned} \tilde{\sigma }(x,Q^2)=F_2(x,Q^2)-\frac{y^2}{1+(1-y^2)}F_L(x,Q^2)\;. \end{aligned}$$

As much of the deterioration in fit quality with decreasing $Q^2_\mathrm{min}$ seems to occur due to a general tendency of the fit to overshoot the HERA neutral current data at highest *y* and low *x* and $Q^2$, the region where the $F_L$ contribution is most important, we will first consider corrections to the $F_L$ theory prediction, before commenting on $F_2$. Motivated by the possible contribution of higher-twist corrections, we consider the very simple possibility

3 $$\begin{aligned} F_L^{(1)}(x,Q^2)=F_L(x,Q^2)\left( 1+\frac{a}{Q^2}\right) \;. \end{aligned}$$

Allowing the parameter *a* to be free and performing a refit, we find a reduction in $\Delta \chi ^2=24$ in the default ($Q^2_\mathrm{min}=2\,\mathrm{GeV}^2$) NNLO fit (and very similar at NLO), with quite a large value of $a=4.30\,\mathrm{GeV}^2$. As this correction will be concentrated in the lower $Q^2$ region we may expect this to affect the trend observed in Figs. 4 and 5 with $Q^2_\mathrm{min}$. In Fig. 6 we show the $\chi ^2/\mathrm{dof}$ with (3) applied by the dashed curves, and we compare with the curves of Fig. 4. The effect is significant, flattening the behaviour essentially entirely. We notice, however, that for the highest $Q^2_{\min }$ considered, i.e. $Q^2_{\min }=10~\mathrm GeV^2$, the $\chi ^2$ obtained with the PDFs and $F_L$ corrections for $Q^2_{\min }=2~\mathrm GeV^2$ can be marginally higher than for the fits obtained for $Q^2_{\min }=2~\mathrm GeV^2$ without the $F_L$ correction. It we perform a refit for each value of $Q^2_{\min }$ then, as in Sect. 5, the improvement in fit quality is minimal, but this feature for $Q^2_{\min }=10\,\mathrm GeV^2$ is removed, and for this higher cut the preferred $F_L$ correction is smaller.

To get a clearer picture, we can look at the effect on the neutral current data/theory comparison. This is shown in Fig. 7 with and without this correction applied. As seen in the left-hand plots there is a tendency to overshoot some of the highest *y* points, and while this is not eliminated entirely for all points by the correction, some tightening of the data/theory is evident and the scatter is more consistent with fluctuations. It is worth pointing out that some of the improvement in $\chi ^2$ actually comes from a reduction in the shift in systematic uncertainties that is required to achieve the optimal fit, which cannot be seen from these figures. It is noticeable that with the correction there is less shift in data relative to theory related to some of the correlated systematics that affect mainly the low *x* and $Q^2$ data, e.g. procedural uncertainty $\delta _1$. Finally we show in Fig. 8 the effect this correction has on the PDFs obtained from the fit when it is included. These changes are seen to be very small, in particular for the global fit. The change in the light sea for the HERA data only fit is due simply to a reshuffling of quarks between different flavours, which is not constrained in this type of fit. In practice the strange quark fraction increases.

In addition to a correction to $F_L$, we may also consider the effect on $F_2$. To do this we consider, as in [23, 24], a further correction

4 $$\begin{aligned} F_2(x,Q^2) \rightarrow F_2(x,Q^2)\left( 1+\frac{a_i}{Q^2}\right) , \end{aligned}$$

where the $a_i$ correspond to $i=1,6$ bins in *x*, all below $x=0.01$, and are left free in the fit. This results in a small additional reduction of $\Delta \chi ^2=10$ in the global fit, but with almost no effect at all on the comparison to the HERA data. Similarly it makes little difference in the HERA data only fit. It therefore appears that at the current level of accuracy the fit does not require any further corrections to $F_2$. Another possibility we consider is an additional $\propto 1/Q^4$ correction to $F_L$: this gives a very small further reduction of $\Delta \chi ^2=5$, with no significant influence on the behaviour with $Q^2_\mathrm{min}$.

While it may be tempting to interpret the above result solely in terms of evidence for higher-twist corrections, it is important to emphasise that the contribution from $F_L$ is only significant at high $y=Q^2/sx$, and thus such a lower $Q^2$ correction is strongly correlated with low *x*. Indeed, if we instead try the correction

5 $$\begin{aligned} F_L^{(1)}(x,Q^2)=F_L(x,Q^2)\left( 1+\frac{\alpha _S(Q^2)}{4\pi }\frac{b_1}{x^{b_2}}\right) \;, \end{aligned}$$

we find a reduction in $\Delta \chi ^2=28$ with $b_1=0.014$ and $b_2=0.82$. However, as at fixed *y* we have $x \propto Q^2$, the power of $b_2 \lesssim 1$ in combination with the slow falling of $\alpha _S$ with $Q^2$ leads to the correction (5) being effectively $\sim 1/Q^2$ for fixed *y*, i.e. consistent with (3).

Finally, we note that detailed examination of data against theory show that the theory predictions at high $Q^2$ and high *y* show a tendency to undershoot the data, that is, the opposite trend to the low $Q^2$ case; this means that for positive $b_1$ a smaller value of $b_2$ in (5) causes problems as it gives a negative correction to the cross section over a wide range of *x* values, whereas the high value of $b_2$ means the effect of the corrections is very much concentrated at small *x*, i.e. only being significant for HERA data for small $Q^2$. Indeed, if we try a $Q^2$ independent correction

6 $$\begin{aligned} F_L^{(1)}(x,Q^2)=F_L(x,Q^2)\left( 1+c_1x^{c_2}\right) , \end{aligned}$$

then the best fit in fact results in an improvement of $\Delta \chi ^2=13$, with $c_1=-1.97$ and $c_2=0.42$. This behaviour leads to a smaller predicted $F_L$, but has its main effect on high *y* data at higher *x* and therefore higher $Q^2$, reducing the tendency of the theory to undershoot the data for the reduced cross section. Taking the sum of (3) and (6) allows an improvement in both the lower and the higher $Q^2$ regions, and it gives a reduction of $\Delta \chi ^2=42$, with $a=5.3\,\mathrm{GeV}^2$ and $c_1=-0.71$, $c_2=0.19$, with *a* being somewhat higher than in the fit with only the $1/Q^2$ correction, consistent with there being some influence from the second term on the lower $x,Q^2$ region.

Hence, the ideal overall correction for $F_L$ is an increase at low *x* and $Q^2$, of higher-twist type, consistent with the tendency for PDF predictions to undershoot the $F_L$ extraction from [25] for $Q^2 < 10~\mathrm GeV^2$, but a reduction at higher *x* and $Q^2$. There are various possible mechanisms where the value of $F_L$ obtained can be modified: the basic power-like higher-twist type of correction explicitly considered; the effects of absorptive corrections to evolution at small *x* and $Q^2$; more general saturation corrections; and resummations of $\alpha _S\ln (1/x)$ terms in the perturbative series. A full study of these is beyond the scope of the present article. Here we simply produce a parametric means of solving the most clear problem in the fit quality for the HERA data.

## Conclusions

We have examined the impact of the final HERA combination of inclusive cross section data presented in [15]. We notice that we already predict these data very well with MMHT 2014 PDFs, particularly at NNLO, and consequently their inclusion leads to very little impact on the central value of the MMHT2014 PDFs. The data do reduce the uncertainty in the PDFs, mainly the gluon, though this is more noticeable in the uncertainty for predictions of benchmark LHC cross sections than in PDF plots, with the uncertainty on Higgs production via gluon fusion being reduced to about 90 % of the previous uncertainty. PDFs obtained from a fit to only the HERA combined data can vary significantly from those from the global fit for some PDFs, but most, including the gluon and down distributions, are similar to the global fit. There is very little constraint on antiquark flavour decomposition. The combined HERA data do seem to prefer a larger up quark above $x=0.2$, and this results in a fit quality for $e^-$ charged current data in a HERA data only fit which is not reproducible in the global fit (though NNLO is better than NLO). We also confirm the result in [15] that the fit quality improves with increasing $Q^2_{\min }$ (though our effect is smaller), and we show that most of this effect is obtained just by changing the cut on the HERA data in the comparison, with little extra contribution when refitting is performed with the raised cut. We note that this $Q^2_{\min }$ behaviour can cured by the addition of a positive “higher-twist” like correction to $F_L$ and that this is more effective than modifications to $F_2$. Small further improvements can also be achieved at higher $Q^2$ by negative corrections to $F_L$ in this region. These corrections result in extremely little change in PDFs obtained from the fit.

Overall we conclude that the current PDFs, with very minor modifications, work extremely well for the final HERA data. The central values of the PDFs are changed very little by the data, even if corrections are added to the theory to improve the fit quality. The data have an impact on uncertainties of PDFs obtained in the global fit, but very largely due to an improvement in the gluon uncertainty. LHC cross sections sensitive to this can have a reduction in uncertainty to about $90~\%$ of their previous values. We do not deem this to be a significant enough effect to warrant an immediate new update of PDFs – there is an “uncertainty on the uncertainty” which is very likely of this order. Instead we prefer to wait for a more substantial update which will include the effects of e.g. full NNLO jet cross sections, NNLO corrections to differential top distributions [26], and the inclusion of significantly more precise, varied, and higher energy LHC data sets.

## Footnotes

1 Initial results were presented in [16] and similar results were also found in [17].

2 The definition of $\chi ^2$ for the HERAPDF2.0 fit is not identical. However, this should be a very small effect.
